# Hepatoprotective Effect of Herb Formula KIOM2012H against Nonalcoholic Fatty Liver Disease

**DOI:** 10.3390/nu7042440

**Published:** 2015-04-02

**Authors:** Hwayong Park, Youn-Hwan Hwang, Dong-Gun Kim, Jongwook Jeon, Jin Yeul Ma

**Affiliations:** KM-Based Herbal Drug Development Group, Korea Institute of Oriental Medicine, Yuseong-daero 1672, Yuseong-gu, Daejeon 305-811, Korea; E-Mails: uofaz@kiom.re.kr (H.P.); hyhhwang@kiom.re.kr (Y.-H.H.); kite79@kiom.re.kr (D.-G.K.); lovejnj@kiom.re.kr (J.J.)

**Keywords:** nonalcoholic fatty liver disease, fatty acid, lipid, herbal medicine

## Abstract

Nonalcoholic fatty liver disease (NAFLD) is a hepatic ailment with a rapidly increasing incidence due to dietary hypernutrition and subsequent obesity. Fatty liver disease can lead to steatohepatitis, fibrosis, cirrhosis, and even cancer, which is associated with various complications. Discovering effective natural materials and herbs can provide alternative and complementary medical treatments to current chemical pharmaceuticals. To develop an effective natural agent for NAFLD, we formulated a combination of four herb mixtures (KIOM2012H) and observed lipid-lowering efficacy. The inhibitory effects of KIOM2012H on free fatty acid-induced lipid accumulation, triglyceride contents, and gene expressions were analyzed in HepG2 cells. Using high fat diet-fed mice, body weight changes, gross liver appearances, hepatic triglyceride contents, and gene expressions were evaluated. KIOM2012H dose-dependently inhibited lipid accumulation and gene expressions involved in lipogenesis and related regulators. Experimental animals also showed a decrease in body weight changes and lipid-associated physiological parameters. This study shows that KIOM2012H has an alleviating effect on fatty acid and lipid accumulation, and therefore can be applied for development of new therapeutic pharmaceuticals for treatment of NAFLD using natural products and herbs.

## 1. Introduction

NAFLD is the most common liver disease involving fat deposited in hepatocytes, and is now becoming one of the worrisome diseases threatening human health, especially in developed countries, with accompanying obesity, cardiovascular and metabolic disease morbidity. It can be developed due to diverse causes such as nutrients, drugs, toxins, metabolites, and other different materials, infections, or diseases [1]. Various phenotypes of liver disease spectrum, such as steatosis, steatohepatitis, fibrosis, cirrhosis, and cancer are diagnosed clinically [2]. Epidemiological reports show that NAFLD is prevailing elsewhere. National Health and Nutrition Examination Surveys data shows a steady increase of NAFLD as a major cause (over 75%) of chronic liver disease in the United States alone [3]. In addition, 13% of children and adolescents between the ages of 2 and 19 years present fatty liver, with the highest prevalence (38%) in obese children [4]. The prevalence of NAFLD cannot be strictly defined and varies among different countries. Generally, about 16% of global normal individuals without metabolic symptoms present NAFLD, but its prevalence increases sharply when they have obesity, diabetes, hyperlipidemia, or are over 60 [5].

The liver is a major internal organ and the principle regulator for maintaining metabolic homeostasis with nutritional materials like lipid and proteins. Typically, it is clinically diagnosed as a fatty liver if excessive fat is more than 5% of liver weight, and more advanced NAFLD can occur by any means in the absence of alcohol intake. Diet, glucose and adipose tissues can also provide liver fatty acid to be utilized for synthesis of triglyceride. Generally NAFLD is known to be caused by accumulated lipids from the above nutrient factors and biological tissues [6]. Clinical research on overweight-obese subjects reported that imbalance between apoptosis and anti-apoptosis is also an important factor affecting hepatocytes and NAFLD [7]. Recently, mitochondrial dysfunction has been revealed as related to NAFLD pathogenesis by research showing that it affects lipid metabolism in the liver, reactive oxygen species (ROS) production, cytokines and apoptosis [8]. Apoptotic cell damage is associated with and affected by mitochondrial membrane depolarization and cytochrome c release [9]. Moreover, *de novo* lipogenesis and reduced lipid clearance in the liver also contributes to hepatic steatosis and lipid-related impaired liver condition [10].

Major symptoms of NAFLD appearing in traditional medicine are anorexia, chronic fatigue, abdominal pain, intestinal convulsion, and nausea. We thoroughly reviewed literature from traditional medicine and selected four herbs *Arctium lappa* (burdock), *Glycyrrhiza uralensis* (licorice), *Magnolia officinalis* (magnolia), and *Zingiber officinale* (ginger), which have been frequently applied in prescription for treatment of the liver-related diseases and symptoms above. According to recent reports and studies, the root of *Arctium lappa* is used as food material and the seeds are known to have antioxidant, antitumor, and anti-inflammation bioactive properties through its representative compound arctigenin [11,12,13]. *Glycyrrhiza uralensis* is the most widely and frequently used herb in traditional medicine as it is an essential component in almost all herbal prescriptions. In addition to various well-known antiallergic [14], anticarcinogenic [15], antiviral [16], and anti-inflammatory properties [17], the most common use is prescriptions for liver disease [18]. *Magnolia officinalis* is also one of the most frequently used herb for liver diseases, especially fatty liver disease, with its attenuating activity of FFA-induced lipogenesis [19]. More recent reports have indicated that the symptoms of fatty liver disease can be improved by *Magnolia officinalis* extract via suppression of tumor necrosis factor α (TNF-α), superoxide anion, and sterol regulatory element-binding transcription factor 1c (SREBF1c) [20]. There is a large number of studies on the role of *Zingiber officinale* in the regulatory mechanisms underlying cholesterol [21], circulating lipids [22], and low-density lipoprotein oxidation [23].

In this study, we formulated KIOM2012H, a combination of four herbs, which is known to have an effect on liver-associated diseases in traditional medicine. Results from *in vitro* cell-based study show the attenuating efficacy with regard to lipid accumulation and its related gene expressions suppressed without cytotoxicity. Additional *in vivo* HFD-fed animal experiments revealed livers with reduced lipid accumulation, weight, and gene expressions in liver. Taken together, the results can be seen as evidence for the potential use of herbs in modern health care and application in complementary and alternative medicine as anti-NAFLD drugs.

## 2. Experimental Section

### 2.1. Herb Materials and Preparation of KIOM2012H

Four medicinal herb materials, *Arctium lappa*, *Glycyrrhiza uralensis*, *Magnolia officinalis*, and *Zingiber officinale,* were employed for KIOM2012H formulation (Table 1). The medicinal herbs were purchased from local vendor Hyundai Herb (Youngchun, Korea), and identified by an expert herbalist to determine that they were the correct species and parts to be used. The combined mixture of four herbs (total 30 g) was immersed in 1 L of distilled water (DW) for 1 h at room temperature to enhance extraction yield, and boiled for 3 h. The crude extract was then filtered (Whatman filter paper #1), lyophilized in a freeze dryer, and stored at −20 °C until use. Lyophilized dry weight of extract was 2.887 g (yield 9.62%). The extract was dissolved in Dulbecco’s phosphate buffered saline (DPBS), centrifuged (13,000 rpm, 10 min, 4 °C) to separate any insoluble, and filtered supernatant through 0.2 μm syringe filter. A voucher specimen of the lyophilized dry extract was deposited in the herbarium of KM-Based Herbal Drug Development Group, Korea Institute of Oriental Medicine (registration No. KIOM2012H).

**Table 1:** Medicinal herbs included in KIOM2012H.

Scientific Name	Family Name	Latin Name	Source	Amount (g)
*Arctium lappa* Linne	Compositae	Arctii Semen	China	9.0
*Glycyrrhiza uralensis* Fischer	Leguminosae	Glycyrrhizae Radix	China	7.5
*Magnolia officinalis* Rehder & Wilson	Magnoliaceae	Magnoliae Cortex	China	6.0
*Zingiber officinale* Roscoe	Zingiberaceae	Zingiberis Rhizoma	Korea	7.5
Total				30.0

### 2.2. Cell Culture

HepG2 cells were obtained from the American Type Culture Collection (Manassas, VA, USA) and maintained in a humidified 5% CO_2_ incubator at 37 °C with 1:1 mixture of Dulbecco’s modified Eagle’s medium and F-12 nutrient (50:50, Life Technologies, Waltham, MA, USA) supplemented with 1% penicillin/streptomycin antibiotic mixture (Gibco, Grand Island, NY, USA) and 10% heat inactivated fetal bovine serum (Gibco, USA). FFA mixture (sodium salts of oleate and palmitate, Sigma-Aldrich, St. Louis, MO, USA) was prepared with bovine serum albumin (BSA), and finally supplemented in BSA-free culture media with concentration of 1%. Cultured cells were used in experiments when they reached 75% confluency.

### 2.3. High Performance Liquid Chromatography (HPLC) Analysis of KIOM2012H

To identify each herb contained in KIOM2012H, HPLC analysis was conducted with standard compounds using HPLC-DAD system Elite Lachrom, composed of a l-2130 pump, l-2200 auto sampler, l-2350 column oven, and l-2455 photodiode array UV/VIS detector (Hitachi High-Technologies, Tokyo, Japan). Separation column RStech OptimaPak C18 (250 × 4.6 mm, I.D. 5 μm, Yale Tech, Guri, Korea) was used at 40 °C, and chromatographic data were processed using EZchrom Elite software (Version 3.3.1a). Injected specimens (10 μL) were separated in the mobile phase composed of 0.1% trifluoroacetic acid (A) and 100% acetonitrile (B) with gradient elution and flow rate of 1.0 mL/min. The standard compounds (liquiritin, glycyrrhinzin, arctiin, matairesinol, arctigenin, 6-gingerol, and magnolol) and lyophilized KIOM2012H powder were prepared by dissolving in 60% methanol. HPLC were performed as described previously [24].

### 2.4. Cell Viability Assay

Cytotoxicity of KIOM2012H was examined using methylthiazolyldiphenyl-tetrazolium bromide (MTT). HepG2 cells were seeded in 48-well plate (6 × 10^4^ cells/well). After 18 h starvation with serum-free medium, the cells treated with different concentrations of KIOM2012H, and incubated for 24 h. Following incubation, 20 μL of MTT solution (5 mg/mL, USB, Austria) was added to each well and incubated at 37 °C for 4 h. Medium was removed, cells were washed with DPBS, and 100 μL of DMSO was added to each well and gently agitated for 5 min to dissolve precipitated formazan. Absorbance at 570 nm was measured in a microplate spectrophotometer (BD Biosciences, Franklin Lakes, NJ, USA).

### 2.5. FFA-Induced Lipid Accumulation and Measurement of Intracellular Triglyceride Content

HepG2 cells were treated with various concentrations of KIOM2012H together with 1 mM FFA for 24 h. Hepatic triglyceride contents were measured using commercial kit Cleantech TG-S (Asan Pharm, Korea) following the manufacturer’s instructions. In brief, specimens were mixed with enzyme mixture and incubated at 37 °C for 10 min. Colorimetric absorbance was determined at 550 nm with a microplate spectrophotometer. Triglyceride contents were shown as amount of triglyceride over total cellular protein, which was determined using a BCA protein assay kit (Thermo Scientific, USA).

### 2.6. Nile Red and Oil Red O Staining

Fluorescent hydrophobic dye Nile Red staining was use to specifically stain intracellular lipids [25,26]. HepG2 cells were treated with KIOM2012H together with 1 mM FFA for 24 h. Cells were washed twice by DPBS and incubated with Nile Red (0.75 μg/mL in DMSO) at room temperature for 15 min. To prevent photobleaching of the dye, plates were incubated in a dark place. Plates were then subjected to a fluorescence spectrophotometer (SpectraMax M2, Molecular Devices, Sunnyvale, CA, USA) with excitation and emission wavelengths of 485 nm and 572 nm, respectively. For Oil Red O staining, cell were seeded to a 48-well plate and pretreated in different concentrations of KIOM2012H for 1 h, followed by 1 mM FFA mixture (0.5 mM oleic acid/0.5 mM palmitic acid at 1:1 ratio) treatment for 16 h, DPBS washing, and 10% formalin fixation for 1 h or longer. After fixation, cells were washed with 60% isopropanol, dried, and stained with Oil Red O solution for 10 min at room temperature. Cells were washed four times with distilled water to remove supernumerary unstained staining solution and were examined under the light microscope. After observed the lipid droplet, 100% isopropanol was added to each well and the intensity of 520 nm absorbance was measured with a spectrophotometer for quantification of Oil Red O content. Seeded cell numbers of culture for Nile Red and Oil Red O were the same as 6 × 10^4^ cells/well.

### 2.7. RNA Isolation and qRT-PCR

Total RNA from HepG2 cells and mouse livers was extracted using Tri-reagent (Sigma-Aldrich, St. Louis, MO, USA). cDNAs were synthesized using High-Capacity cDNA Reverse Transcription Kit (ABI, Waltham, MA, USA). Reverse-transcription and qRT-PCR were performed to detect relative mRNA expression using TaqMan probes (Thermo Scientific, USA) and TaqMan Universal Master Mix, as described previously [27], in ABI 7500 Real-Time PCR System (ABI, USA). Seeded cell number in culture for RNA isolation was 7 × 10^5^ cells/dish (60 mm). Gene expressions of SREBF1c, fatty acid synthase (FASN), and CD36 (cluster of differentiation 36, *i.e.*, fatty acid translocase FAT) were analyzed in HepG2 cells, and FASN, CD36, cell death-inducing DFFA-like effector C (CIDEC, alternatively, fat-specific protein FSP27), Perilipin 2 (PLIN2, alternatively, adipose differentiation-related protein ADRP), and peroxisome proliferator-activated receptor-γ coactivator 1α (PPARGC1A) were analyzed in livers from experimental animal. Expression levels of Ubiquitin C (UBC) were used for nomalization.

### 2.8. Measurement of Fatty Acid Uptake

After treatment with or without KIOM2012H (500 μg/mL) for 24 h, cells were washed with DPBS and further incubated in DPBS containing 0.2% BSA and 1 μΜ BODIPY-labeled fatty acid C16 (Life Technologies, USA) for 30 min at 37 °C. Cells were washed again twice with cold DPBS containing 0.2% BSA to remove residual cell surface-bound BODYPY and fluorescence intensities were measured by flow cytometry (FACSCalibur, BD Biosciences, USA) with excitation and emission wavelengths of 503 nm and 512 nm, respectively.

### 2.9. In Vivo Animal Experiment

The experimental animals, C57BL/6 male mice (7 weeks old), were obtained from Orient Bio Inc. (Sungnam, Korea) and acclimated for 1 week in our animal laboratory. Animals were divided into 6 groups (13 in each group) by their body weight and maintained under the standard conditions of temperature 22.5 ± 0.5 °C, humidity 42.6% ± 1.7%, lighting 12 h (8:00–20:00, 290 Lux), and ventilation 10–15 times per hour, for the 14-week experimental period. High fat diet (HFD, 60%, Research Diets Inc., New Brunswick, NJ, USA) and water were provided *ad libitum*. KIOM2012H was dissolved in distilled water and distilled water was used as vehicle control. KIOM2012H was administered once a day orally at 3:00 pm, and body weight was measured once a week. At the end of the experimental period, mice were euthanized by CO_2_ asphyxia and examined for any abnormal alterations, followed by removing livers for further analysis. All the animal experiments were conducted in accordance with the “Guide for the care and use of laboratory animals (NIH)” and underwent deliberation from the “Institutional Animal Ethics Committee of Korea Institute of Korea Institute of Oriental Medicine” (Review Document No. 12-108, Review Date 31 October 2012).

### 2.10. Statistical Analysis

All the experimental values obtained were expressed as mean ± SE. Multiple group comparisons were performed with one-way analysis of variance (ANOVA) followed by Dunnett’s test, while two group comparisons were performed with Student’s *t* test. The results were considered statistically significant when the *p* value was less than 0.05 or 0.01.

## 3. Results

### 3.1. HPLC Fingerprint of KIOM2012H

**Figure 1:**
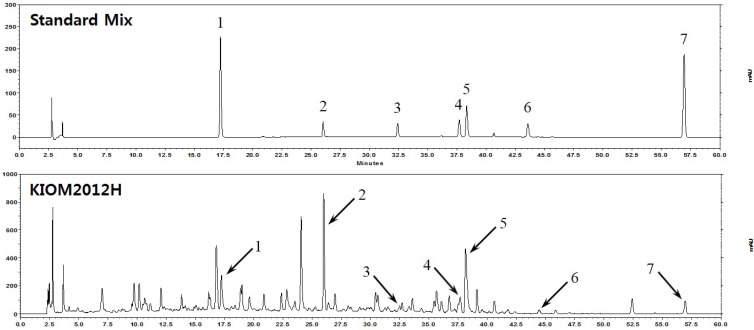
HPLC chromatogram of standard markers and KIOM2012H. Standard markers of liquiritin (1), arctiin (2), matairesinol (3), arctigenin (4), glycyrrhinzin (5), 6-gingerol (6), and magnolol (7) are shown in the above. HPLC chromatogram of KIOM2012H is shown in the bottom with arrows corresponding each standard marker.

HPLC was conducted with standard markers to identify which exact herbs are included in KIOM2012H, composed of multiple herbs, and for comparative quantification of each component. Figure 1 shows a typical chromatogram of standard mix and representative chromatogram of each peak from KIOM2012H, corresponding to each standard. By this analysis, four herbs composing KIOM2012H were identified as it contains seven standards: liquiritin, arctiin, matairesinol, arctigenin, glycyrrhizin, 6-gingerol, and magnolol.

### 3.2. Cytotoxicity of KIOM2012H

**Figure 2:**
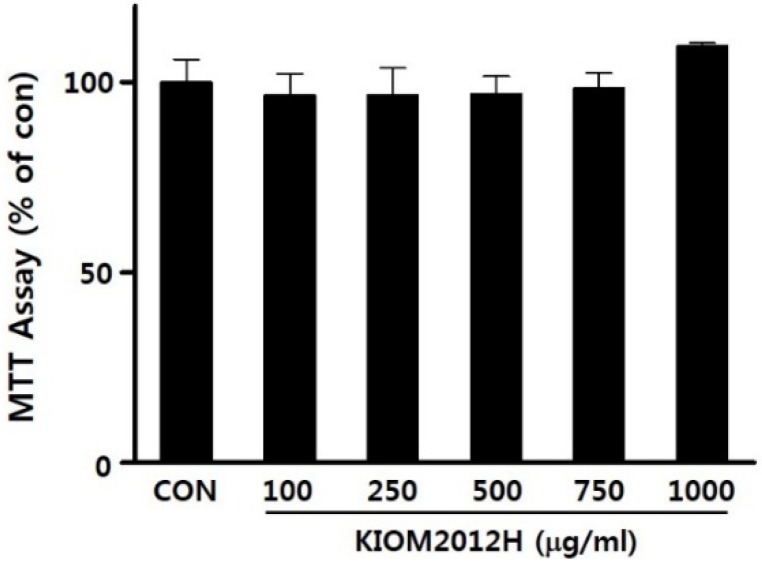
Effect of KIOM2012H on HepG2 cell viability treated with different concentrations of KIOM2012H for 24 h. Data represent mean ± SE of three independent experiments for accuracy.

The cytotoxicity of KIOM2012H on HepG2 cells was examined by MTT assay. KIOM2012H treatment for 24 h with concentrations of up to 1000 μg/mL showed no significant effects on HepG2 cell viability (Figure 2) and *in vivo* mouse acute toxicity. Higher doses (1500 and 2000 μg/mL) of KIOM2012H reduced cell viability up to 89.5 ± 9.9 and 77.7 ± 8.0 μg/mL, respectively.

### 3.3. Effect of KIOM2012H on FFA-Induced Lipid Accumulation in HepG2 Cells

**Figure 3:**
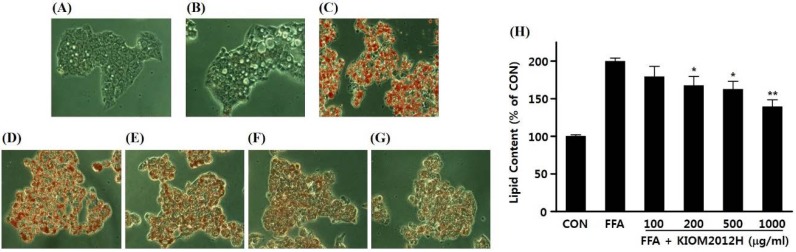

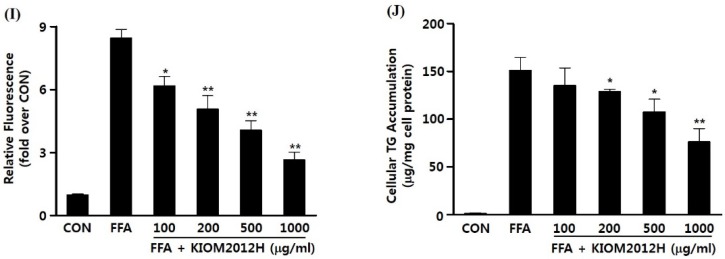
Inhibition of FFA-induced lipid accumulation (microscopic image), lipid contents, and triglyceride contents by KIOM2012H in HepG2 cells. Untreated (**A**); 0.1% BSA treated control (**B**); 1 mM FFA treated (**C**); and 1 mM FFA cotreated with different concentrations of KIOM2012H (**D**–**G**) shows dose-dependent decrease of Oil Red O stained lipid via microscope imaging (400×), and also via quantitative analysis (**H**); Intracellular lipid accumulation was also observed via Nile Red staining (**I**) and triglyceride contents were measured (**J**). Data represent mean ± SE of three independent experiments. Statistical differences from FFA are indicated by asterisks (* *p* < 0.05, ** *p* < 0.01).

HepG2 cells were used to identify the inhibiting effect of KIOM2012H on FFA-induced hepatic cellular steatosis *in vitro*. Lipid accumulation was induced by supplementation of FFA, palmitic acid, and oleic acid, to make hepatocytes supply excessive FFA. Intracellular lipids were stained with Oil Red O and observed under the phase-contrast microscope. In contrast with untreated cells (Figure 3A) and BSA-only treated cells (Figure 3B), FFA treated cells showed significant Oil Red O stained lipids accumulated. Pretreatment of KIOM2012H on HepG2 cells inhibited lipid accumulation dose-dependently (Figure 3D–G), and its corresponding quantitative cellular lipid content was shown (Figure 3H). The inhibition effect of KIOM2012H on lipid accumulation was further confirmed by Nile Red staining (Figure 3I) and also by quantification of cellular triglyceride contents (Figure 3J), which were shown to dose-dependently decrease lipids in the cells.

### 3.4. KIOM2012H Inhibited Gene Expression and Lipid Uptake

**Figure 4:**
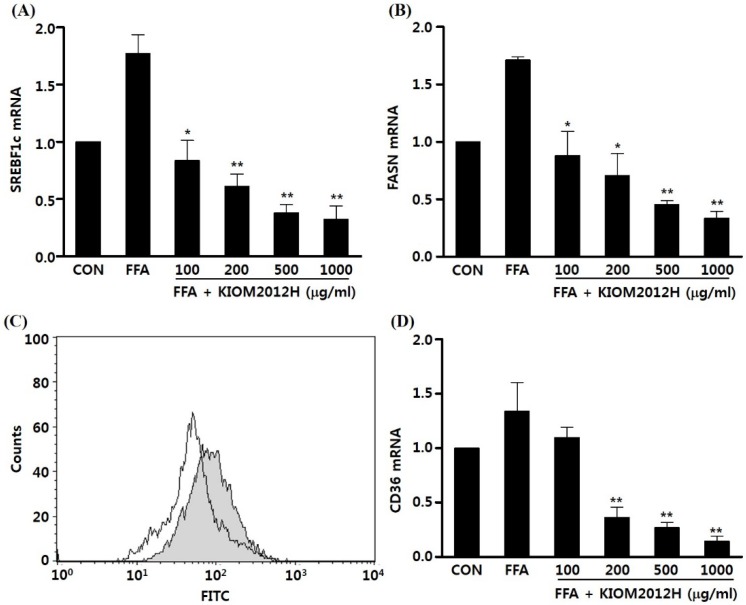
KIOM2012H attenuates lipogenic mRNA gene expression and fatty acid import. HepG2 cells were treated with 1 mM FFA in the absence or presence of various concentrations of KIOM2012H for 24 h. qRT-PCR analysis shows decreased mRNA levels of SREBF1c (**A**) and FASN (**B**); Cells were incubated with the BODIPY-labeled fatty acid C16 in the absence (gray) or presence (white) of KIOM2012H and fluorescence was measured by flow cytometry (**C**); Fatty acid translocase CD36 gene expression was also dose dependently down regulated by KIOM2012H (**D**). Data represent mean ± SE of three independent experiments. Statistical differences from FFA are indicated by asterisks (* *p* < 0.05, ** *p* < 0.01).

For detailed molecular biological analysis of genes involved in lipid accumulation and related inhibiting activity of KIOM2012H *in vitro* HepG2 cells, mRNA expression levels of SREBF1c and FASN were observed using quantitative real-time PCR. FFA highly increased expression of both SERBF1c and FASN compared to the BSA-only-treated control, which was again dose-dependently decreased by KIOM2012H, even lower than the control (Figure 4A,B). CD36 (leukocyte differentiation antigen or FAT) was analyzed using mRNA expression using qRT-PCR. CD36 is known as a membrane-bound glycoprotein and functions as a cell adhesion molecule and receptor for low density lipoprotein. To identify whether FFA uptake is conducted by this transporter protein, cells were incubated with BODIPY-labeled FFA and analyzed by flow cytometry. BODIPY-labeled FFA uptake (Figure 4C gray) was significantly decreased with treatment of KIOM2012H (Figure 4C white). Like the above two genes, CD36 gene expression was also dose-dependently inhibited by KIOM2012 treatment when comparing the control and FFA-treated group (Figure 4D). These result demonstrate that reduced CD36 gene expression and the subsequent altered membrane-bound transporter protein contributed to this limited FFA uptake into the cells.

### 3.5. KIOM2012H Effects in HFD-Fed Mice

**Figure 5:**
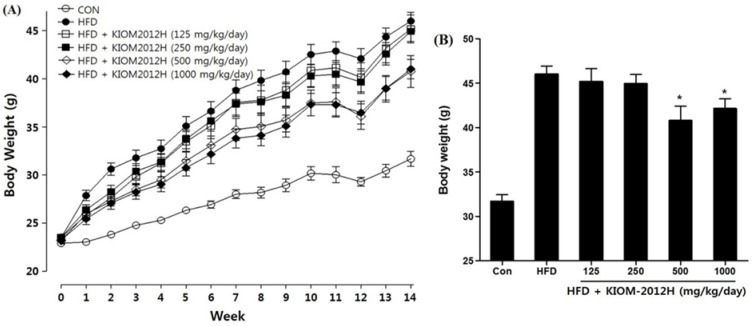
The changes in body weight during all the experimental period (**A**) and at necropsy (**B**) were dose-dependently decreased in HFD-fed mice (*n* = 13) as well as with KIOM2012H. Statistical differences from HFD are indicated by asterisks (* *p* < 0.05, ** *p* < 0.01).

Experimental animal C57BL/6 mice were supplied with HFD and KIOM2012H for 14 days, and there were no specific clinical signs, behavioral alterations, or acute toxicity. Body weight increase in HFD-fed animals was higher than that of the normal control group, but it was dose-dependently decreased by KIOM2012H (Figure 5A). Although KIOM2012H treatment tends to decrease relative liver weights, there was no significant increase in relative liver weights between the control and KIOM2012H-treated groups (Figure 5B).

**Figure 6:**
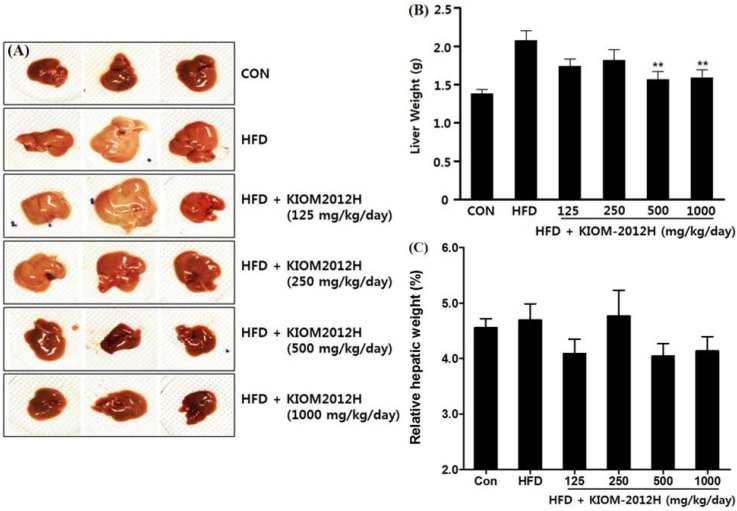

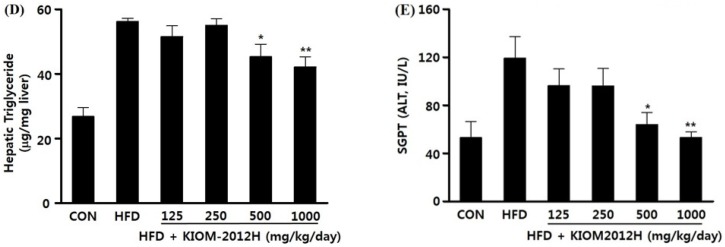
Livers from HFD-fed mice (*n* = 13) show hypertrophy (**A**); absolute liver weight (**B**); relative liver weight (**C**); hepatic triglyceride content (**D**); and serum SGPT level (**E**); which were closer to normal for KIOM2012H. Statistical differences from HFD are indicated by asterisks (* *p* < 0.05, ** *p* < 0.01).

The external appearance of HFD-fed livers included hypertrophy and a yellowish color (from accumulated fat), but KIOM2012H-treated livers were recovered and found to be near normal in terms of their color, size, and weight, in a dose-dependent manner (Figure 6A–C). Hepatic triglyceride contents and serum glutamic-pyruvic transaminase (SGPT) levels were also dose-dependently lowered (Figure 6D,E).

**Figure 7:**
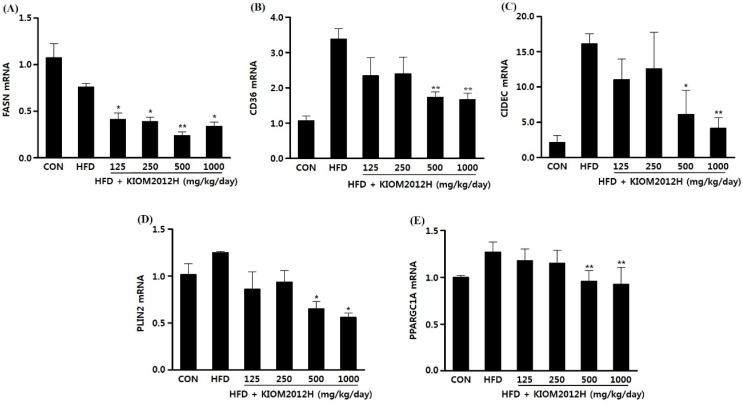
Relative expression levels of genes involved in FASN (**A**); CD36 (**B**); CIDEC (**C**); PLIN2 (**D**); and PPARGC1A (**E**) observed in mice liver nucleic acid (*n* = 13). Data represent mean ± SE. Statistical differences from HFD are indicated by asterisks (* *p* < 0.05, ** *p* < 0.01).

Expressions of fatty liver disease-related genes in liver were analyzed by qRT-PCR, in which FASN and CD36 were down-regulated according to the increasing dose of KIOM2012H in a similar way to HepG2 cells (Figure 7A,B). CIDEC, PLIN2, and PPARGC1A gene expressions were also lowered close to or even much lower than the control by KIOM2012H treatment in HFD-fed animal liver tissues (Figure 7C–E).

## 4. Discussion

NAFLD is considered to be the most common liver disease in Western countries and is increasingly being diagnosed worldwide. It encompasses a wide spectrum of liver disease which includes variable degrees of simple steatosis (fatty liver), nonalcoholic steatohepatitis (NASH) and cirrhosis. Simple steatosis is benign, whereas steatohepatitis is characterized by hepatocyte injury, inflammation and fibrosis, which can lead to cirrhosis [28]. The liver plays a major role in lipid metabolism by absorbing serum FFA and manufacturing, storing, and transporting lipid metabolites. The accumulation of lipids, mainly triacylglycerol (TAG), in hepatocytes is the hallmark feature of the pathogenesis of NAFLD. The increased transport of FFAs into hepatocytes leads to enhanced hepatic *de novo* lipogenesis with excessive hepatic FFA β-oxidation and very low-density lipoprotein (VLDL) export, resulting in hepatic steatosis [29]. Although the disease can be managed by improvement of lifestyle (diet, exercise and weight reduction) and cardiometabolic risk factors via controlling elevated cholesterol, triglyceride, and blood sugar, there are still no drug treatment options for NAFLD.

In this study, to develop an herbal medicine formula for the treatment of NAFLD, we formulated KIOM2012H using four medicinal herbs, which have been used to treat diseases related to the liver in over 120 traditional herbal prescriptions. To evaluate the effect of KIOM2012H on NAFLD, we used both *in vitro* cell models of lipid overloading and *in vivo* dietary models of NAFLD. Our results showed that KIOM2012H treatment significantly reduced lipid accumulation on hepatocytes in the presence of pathophysiologically relevant concentrations of palmitic acid and oleic acid in a dose-dependent manner. Furthermore, morphologic changes were also observed using Oil Red O and Nile Red fluorescence staining, and then quantitative analysis was performed to measure the intracellular triglyceride levels. To exclude the possibility that the inhibitory effect of KIOM2012H on lipid accumulation due to cytotoxicity, a cell viability test was conducted after treatment with various concentrations of KIOM2012H. As a result, we determined the efficacy of KIOM2012H as a lipid-lowering agent for the *in vitro* cellular model of hepatic steatosis without cytotoxicity. To confirm these results in an *in vivo* system, we used a HFD-induced NAFLD mouse model. From this experiment, we found that oral administration of KIOM2012H significantly reduced HFD-induced hepatic hypertrophy, hepatic triglyceride and serum SGPT without specific clinical signs or behavioral alterations. Subsequently, we observed food and water intake, serum triglyceride and blood glucose. However, there was no significant difference for those parameters (data not shown). Therefore, further in-depth studies are needed to clarify the *in vivo* anti-obesity effect of KIOM2012H on FFA uptake, energy expenditure, fat mass, insulin sensitivity, and so on.

From the molecular mechanism studies, we found that increased lipid deposition by FFA treatment induced the mRNA expression of SREBP-1c, which is a major regulatory transcription factor that controls lipogenesis and FAS, an enzyme involved in the metabolism of glucose to fatty acids [30]. Interestingly, KIOM2012H treatment significantly repressed the FFA-induced mRNA expression of SREBF1c, leading to reduced expression of FAS and lipid synthesis. Furthermore, KIOM2012H also repressed the expression of fatty acid translocase CD36, suggesting that KIOM2012 could affect the uptake of FFA. Previous studies showed that CD36 gene expression was increased and correlated with liver fat content in patients with NAFLD [31], and CD36 deletion protected against the development of hepatic steatosis [32,33]; inhibiting the uptake of FFA into the hepatocyte can be affected by the inhibition of CD36 expression. CIDEC, as the name implies, was overexpressed in the cells of nuclei condensed and DNA fragmented, which is a typical apoptotic event [34]. Beside of the apoptotic event, CIDEC was reported as up-regulated and overexpressed during adipogenesis and lipid deposition [35,36]. Our result also corresponds with previous reports, as the expression of CIDEC was immensely high in HFD-fed mouse liver, and in turn dose-dependently down-regulated close to the normal control. PLIN2 and PPARGC1A, known to be involved in adipose tissue and play a pivotal role in glucose and fatty acid metabolism, respectively, were also observed as they were down-regulated by KIOM2012H. Our results from FACS analyses and CD36 expression by treatment of KIOM2012H suggest that the anti-NAFLD effect of KIOM2012H likely results from its inhibition of CD36 gene expression and the consequent decrease in hepatic FFA uptake.

HPLC analysis identified seven main components—arctiin, matairesinol, arctigenin liquiritin, glycyrrhizin, 6-gingerol, and magnolol—included in KIOM2012H. A previous study reported that glycyrrhizin is used clinically for treatment of viral hepatic diseases such as the human immunodeficiency virus and hepatitis C infection [37]. Additionally, it was reported that 6-gingerol and its analogue showed protective effects with respect to the development of metabolic syndrome in mice fed a high-fat diet [38]. Moreover, *Magnolia officinalis,* which contains magnolol as the primary pharmacological component, reverses alcoholic fatty liver by inhibiting the maturation of SREBF1c [20].

## 5. Conclusions

The herb formula KIOM2012H inhibited lipid accumulation in hepatocytes, at least in part, by suppressing lipogenesis and uptake of FFA. Therefore, our study suggests that KIOM2012H may be useful for the prevention and treatment of NAFLD.
